# Does Sugammadex Decrease Length of Stay in Bariatric Surgery? Experience from an MBSAQIP Comprehensive Bariatric Center

**DOI:** 10.1007/s11695-026-08910-z

**Published:** 2026-08-25

**Authors:** Dominick Ricci, Ashton Norris, Ratna Pallapothu

**Affiliations:** Department of Surgery, University of South Carolina School of Medicine, Columbia, USA

**Keywords:** Bariatrics, Foregut, Reversal, Sugammadex, Length of stay

## Abstract

**Background:**

Residual effects from general anesthesia can delay the time until oral intake and ambulation. Reversal with Sugammadex has been shown to improve outcomes and decrease post-operative nausea and vomiting. The benefits amongst the bariatric population have not been ascertained. We hypothesized that the use of Sugammadex for reversal will decrease the length of stay (LOS).

**Methods and Procedures:**

This is a single center retrospective review of 961 patients who underwent elective bariatric surgery from January 2023 to January 2025. Inclusion criteria were any patient undergoing elective bariatric surgery who received either Sugammadex or neostigmine. Our exclusion criteria were any emergent or open procedures. The decision to administer either Sugammadex or neostigmine was up to the discretion of the anesthesiologist. Sugammadex was used in 355 patients and neostigmine for reversal in 606 patients. The primary outcome was the length of stay. Multivariable regression models were used to adjust various variables.

**Results:**

Median LOS was 2 nights in the Sugammadex group versus 3 nights in the neostigmine group. The proportion of prolonged stays (> 2 nights) was 27.9% for Sugammadex (*p* = 0.007) versus 72.1% for neostigmine (*p* = 0.004). In multivariable logistic regression, sugammadex was independently associated with lower odds of prolonged LOS (OR = 0.55, 95% CI 0.37–0.82, *p* = 0.004) while neostigmine was associated with increased odds of prolonged LOS (OR = 1.866, 95% CI 1.23–2.83, *p* = 0.003). Higher complexity procedures and higher BMI were also independent predictors of increased LOS.

**Conclusion:**

Sugammadex use was associated with significantly lower odds of prolonged hospital stay following elective bariatric surgery. Procedure complexity and higher BMI were also independent predictors. Further prospective studies are warranted to confirm these findings.

## Introduction

Obesity continues to be an epidemic in the United States with an estimated 172 million adults being overweight or obese [1]. This epidemic comes with a significant health burden. In 2021, an estimated 335,000 deaths and 11.6 million disability-adjusted life years were attributed to obesity.^1^Accompanying this is an astronomical economic burden with healthcare costs estimated between 260 and 480 billion dollars in 2016. Forecasts do not anticipate any change in the current trajectory with one study estimating by 2050 that two thirds of all adults will be overweight or obese [1]. Despite this, access to metabolic and bariatric surgery remains severely limited. The 2018 position paper by the ASMBS estimates that only 1.1% of patients who qualified for metabolic and bariatric surgery underwent an operation [2].

Surgery remains the most durable treatment for obesity contributing to prolonged remission of a host of other comorbid conditions. ERAS protocols implementation has further improved the efficiency of Bariatric surgery with a faster perioperative recovery. While some institutions are moving towards a same day discharge model, a 2021 study examining the MBSAQIP database found that only 7.4% of patients were discharged on the same day [3]. There is also an increasing trend amongst most insurances towards a push for these complex procedures performed under observational status rather than an inpatient. Same day discharge is an aspirational goal however the criteria have not been established with certainty. Therefore, research into modifiable factors to decrease length of stay is paramount.

Anesthesia teams are rapidly moving towards widespread recommendation and adoption of Sugammadex, a molecule that encapsulates and complexes to aminosteroid neuromuscular blockade agents. Drugs such as rocuronium are then inactivated and acetylcholine can bind to its receptors [4]. The 2023 ASA guidelines for neuromuscular blockade and reversal have a strong recommendation for the use of Sugammadex for all levels of blockade when rocuronium is used. Multiple randomized control trials showed a lower incidence of residual blockade with Sugammadex use compared with neostigmine [5]. There is also a consistent and prompt effect once administered that is not altered by patient BMI [6]. These qualities make it an ideal reversal agent for the bariatric population.

Studies thus far have not examined the effect on the bariatric population. We begin to address that gap in the literature with this retrospective cohort study at a single MBSAQIP accredited comprehensive center over a two-year period.

## Materials and Methods

### Study Design

This is a single center (MBSAQIP accredited comprehensive center), retrospective cohort study of 961 patients undergoing elective bariatric surgery from January 2023 to January 2025. MBSAQIP registry entry was utilized for data collection. Data was further abstracted from the electronic medical record. Institutional review board approval was obtained. This study is reported following the Strengthening the Reporting of Observational Studies in Epidemiology (STROBE) guidelines [7]. The study did not receive any grant or funding from the public, commercial, or not-for-profit sectors.

### Inclusion and Exclusion Criteria

All patients undergoing elective bariatric surgery were identified using the MBSAQIP registry. We included those who received either Sugammadex or neostigmine. Our exclusion criteria were patients who underwent emergent or open procedures.

### Institutional Sugammadex Administration / Extracted Data Points

The decision to administer either Sugammadex or neostigmine was up to the discretion of the anesthesiologist. Surgeons at our institution did not contribute to the decision. The primary outcome was length of hospital stay (LOS), which was defined as prolonged if > 2 days.

### Statistical Analysis

Continuous variables were reported as medians with interquartile ranges (IQRs) and analyzed using Mann–Whitney U tests. Categorical variables were reported as percentages and analyzed using Chi-squared tests. Multivariable logistic regression models were used to adjust various variables. The effect size of predictors was interpreted as the percent change in odds of reinsertion per unit increase, calculated as (OR − 1) × 100 for odds ratios above 1 and (1 − OR) × 100 for odds ratios below 1. Statistical analyses were performed using JASP software (version 0.19.3, University of Amsterdam).

## Results

### Patient Characteristics

A total of 961 patients met the inclusion criteria. For neuromuscular blockade reversal, 355 (36.9%) received Sugammadex and 606 (63.1%) received neostigmine. Medians for the overall cohort were 39.9 years (IQR 17.3) for age, 37.7 kg/m² (IQR 17.8) for BMI, and 97 min (IQR 63) for operative time. Most patients were female (91.2%) and ASA class III (84.8%). Primary procedures included Sleeve gastrectomy (50.9%), Roux-en-Y gastric bypass (35.9%), BPD-DS/Duodenal switch (10.6%), and Single-anastomosis duodenoileostomy with sleeve gastrectomy/SADI-S (2.6%).

### Comparison of Variables

Patients with Sugammadex use had a slightly higher BMI compared to those with neostigmine use (*p* = 0.047), while age and operative duration were similar between groups and not statistically significant. Median LOS was 2 nights in the Sugammadex group versus 3 nights in the neostigmine group. There was an average decrease of 0.6 days between groups. A prolonged length of stay occurred in 172 patients (17.9%), of those 27.9% received Sugammadex and 72.1% received neostigmine.

In univariate analysis, several factors were significantly associated with prolonged LOS. These included reversal agent (neostigmine 20.5%, *p* = 0.004; Sugammadex 13.5%, *p* = 0.007), primary procedure type (*p* < 0.001), conversion/revision surgery (26.5% vs. 16.7%, *p* = 0.010), obesity class (*p* < 0.001), and operative duration (*p* = 0.043). Sex, race, ASA classification, diabetes, GERD, payor status, and robotic assistance were not significantly associated with prolonged LOS in univariate analysis (all *p* > 0.05).

In multivariable logistic regression, adjusting for patient demographics, procedure type, comorbidities, operative duration, and payor status, Sugammadex use was independently associated with reduced odds of prolonged LOS (OR = 0.55, *p* = 0.004). In an additional covariate model, conversely from Sugammadex, neostigmine was associated with higher odds of prolonged LOS (OR = 1.866, *p* = 0.003). Insurance and certain procedure categories were also independent predictors of increased LOS. Those with Medicaid had increased odds of prolonged LOS (OR = 1.632, *p* = 0.022). Higher complexity procedures were associated with prolonged LOS, specifically BPD-DS (OR = 3.206, *p* < 0.001) and conversions or revisions when compared to index bariatric surgery (OR = 2.079, *p* = 0.038). The area under the curve (AUC) for both models was 0.69, indicating modest discrimination.

In sensitivity analyses including operating surgeons as a covariate, the association between reversal agent and prolonged LOS was attenuated and no longer statistically significant (Sugammadex OR 0.80, *p* = 0.31; neostigmine OR 1.26, *p* = 0.29). However, the surgeon was not included in the final model. Also of note, there was a large variation amongst surgeons in prolonged LOS (0-27.2%).

## Discussion

The global health community is experiencing an obesity epidemic with seemingly no end in sight. Obesity rates amongst children and adults are projected to continue to rise significantly [1]. Surgery remains the gold standard and most durable treatment for obesity and its comorbid conditions. When the treatment is known, the next step is to improve access. A paltry 1% of patients who would benefit from surgery undergo this life changing procedure [2]. ERAS protocols and societal guidelines have been created to safely optimize the surgical process with Sugammadex being recommended as the standard of care reversal agent in the latest ASA guidelines [5]. Studies looking into the effect of reversal agent on the bariatric population are lacking, especially with that population steadily growing.

Several studies have investigated the outcomes when comparing Sugammadex and neostigmine due to the observed clinical differences. Sugammadex has been associated with several positive outcomes, including shorter operative room time, decreased postoperative nausea and vomiting (PONV), and shorter time in postoperative recovery. One specific argument against Sugammadex is its increased cost, approximately 5x the cost of neostigmine. The counterargument is the costs saved with improved outcomes, therefore, decreased hospital resource utilization [8].

The distinction is secondary to the respective mechanisms of action on reversal of the neuromuscular blockade (NMB). Sugammadex is a selective inhibitor and acts by binding to the relaxant, encapsulating and inactivating it, and promoting excretion of the formed complex; altogether leading to rapid reversal. Alternatively, neostigmine works competitively and indirectly to reverse NMB by inhibiting acetylcholinesterase and increasing the chance of relaxant displacement, thus reversing NMB. This process is slower and requires a certain amount of spontaneous recovery before truly effective. Furthermore, neostigmine has adverse side effects related to cholinergic mechanism [9]. These fundamental differences in pharmacodynamics explain why Sugammadex is faster, more effective with deep NMB, and has decreased residual paralysis. Residual paralysis also leads to increased risk of ICU admission, with additional consumption of hospital resources increasing cost [10].

The high prevalence of residual NMB with neostigmine reversal is well-documented. The RECITE-US study was a prospective, multicenter trial with 255 patients undergoing abdominal surgery. They found that 64.7% of patients had residual NMB at extubation despite administration of neostigmine. Notably, higher BMI was independently associated with increased odds of residual blockade (OR 1.04 per unit, *p* = 0.043), suggesting that bariatric surgery patients may be particularly vulnerable to incomplete reversal with neostigmine. This finding alone validates our observation that Sugammadex usage was associated with reduced LOS in this high-risk population [11].

The mechanism to explain increased risk of NMB in obese patients is related to pharmacokinetics with increased volume of distribution, thus prolonging excretion and clearance. Additionally, neostigmine may be particularly ineffective due to the need for some amount of spontaneous recovery prior to administration and measures to reverse deep blockade [4]. The RECITE-US study authenticates this by identifying higher BMI as a risk factor for residual NMB [11]. While we did not specifically look at pharmacologic effects, the literature on the mechanism of action supports future investigation in the bariatric population to fully elucidate NMB reversal effect, expanding the variables analyzed or in a prospective study.

The inpatient hospital time and resource utilization saved theoretically lead to decreased LOS, a finding which is elicited by our study. Similar outcomes of Sugammadex association with decreased LOS were noted by Crosby et al. (2025). However, our findings are unique and more specific to this topic, as the primary outcome and conclusion by Crosby et al. were decreased postoperative urinary retention, in addition to a smaller sample size than our study [12].

### Limitations

We acknowledge that this study has several limitations. First, the retrospective, single-center design limits generalizability and introduces potential selection bias. The choice of reversal agent at the discretion of the anesthesiologist could be unmeasured by confounders that might influence both drug selection and outcomes. We did not capture the depth of neuromuscular blockade at reversal, dosing, or postoperative respiratory or other complications. We did not have data on actual train-of-four ratios at extubation or PACU arrival. The proposed mechanism or residual NMB is inferred from the literature but not directly demonstrated in our cohort. Furthermore, prolonged LOS is a surrogate outcome influenced by multiple factors beyond NMB reversal, and we did not capture specific reasons for delayed discharge. Prospective, randomized studies with quantitative neuromuscular monitoring and more specific outcome measures, such as respiratory issues, are needed to confirm these findings.

Finally, the exclusion of surgeons in our final model with a sensitivity analysis negates the significance of our primary outcome. Our rational is validated first by a 2022 JACS study that found patient factors contributed more to LOS than surgeon or hospital factors [13]. Another reason, we have a standardized bariatric protocol in postoperative care that aligns across the four surgeons and is ensured by the team of surgical residents. This implies very little variation in discharge parameters. A final rational, the drug choice was determined by the anesthesiologist, therefore surgeon-level factors should have limited effect on the primary outcome of interest. Including the surgeon would risk overadjustment. A more likely explanation for the large variation in prolonged LOS rates between surgeons is that LOS rates may be associated with differing case complexity or patient selection.

## Conclusion

In this retrospective analysis of 961 patients undergoing elective bariatric surgery, Sugammadex use for NMB reversal was associated with significantly lower odds of prolonged hospital length of stay compared to neostigmine. Our study has one of the largest sample sizes specifically evaluating the bariatric population. These findings suggest that Sugammadex may offer clinical benefits beyond faster reversal times in the bariatric population, potentially through reduction of residual NMB and its associated complications. Prospective, randomized studies are warranted to confirm these findings and evaluate cost-effectiveness in this high-risk population.

## Data Availability

No datasets were generated or analysed during the current study.

## References

[1] Phelps N, Singleton R, Zhou B, et al. Worldwide trends in underweight and obesity from 1990 to 2022: a pooled analysis of 3663 population-representative studies with 222 million children, adolescents, and adults. Lancet. 2024;403:1027–50. 10.1016/S0140-6736(23)02750-2 PMC7615769 38432237

[2] English WJ, DeMaria EJ, Hutter MM, et al. American Society for Metabolic and Bariatric Surgery 2018 estimate of metabolic and bariatric procedures performed in the United States. Surg Obes Relat Dis. 2020;16(4):457–63. 10.1016/j.soard.2019.12.022. 32029370

[3] Bharani T, Tavakkoli A, Tsai TC, Robinson MK, Sheu EG. Safety of Same-Day Discharge after Bariatric Surgery: Retrospective 6-Year North American Analysis. J Am Coll Surg. 2024;238(6):1023–34. 10.1097/XCS.0000000000001052. 38376072

[4] Hagberg CA, Asai T, Heidegger T, Myatra SN, Pandit JJ, Jiang Y. Era of sugammadex: A paradigm shift in airway management. Anesthesiology. 2026;144(4):978–97. 10.1097/ALN.0000000000005828 41805213

[5] Thilen SR, Weigel WA, Todd MM, Dutton RP, Lien CA, Grant SA, Szokol JW, Eriksson LI, Yaster M, Grant MD, Agarkar M, Marbella AM, Blanck JF, Domino KB. American society of anesthesiologists practice guidelines for monitoring and antagonism of neuromuscular blockade: A report by the American society of anesthesiologists task force on neuromuscular blockade. Anesthesiology. 2023;138(1):13–41. 10.1097/ALN.0000000000004379 36520073

[6] Mostoller K, Wrishko R, Maganti L, Herring WJ, van Zutphen-van Geffen M. Pharmacokinetics of Sugammadex Dosed by Actual and Ideal Body Weight in Patients With Morbid Obesity Undergoing Surgery. Clin Transl Sci. 2021;14(2):737–44. 10.1111/cts.12941. PMC7993252 33278332

[7] Von Elm E, Altman DG, Egger M, et al. 28The Strengthening of the Reporting of Observational Studies in Epidemiology (STROBE) Statement: Guidelines for reporting observational studies. Int J Surg. 2014;12:1495–9. 10.1016/j.ijsu.2014.07.013 25046131

[8] Moss AP, Powell MF, Morgan CJ, Tubinis MD. Sugammadex versus neostigmine for routine reversal of neuromuscular blockade and the effect on perioperative efficiency. Proc (Bayl Univ Med Cent). 2022;35(5):599–603. PMID: 35991723; PMCID: PMC9373753. 10.1080/08998280.2022.2079921 PMC9373753 35991723

[9] Chhabra R, Gupta R, Gupta LK. Sugammadex versus neostigmine for reversal of neuromuscular blockade in adults and children: A systematic review and meta-analysis of randomized controlled trials. Curr Drug Saf. 2024;19(1):33–43. 10.2174/1574886318666230302124634. 10.2174/1574886318666230302124634 36861797

[10] Grabitz SD, Rajaratnam N, Chhagani K, Thevathasan T, Teja BJ, Deng H, Eikermann M, Kelly BJ. The effects of postoperative residual neuromuscular blockade on hospital costs and intensive care unit admission: A population-based cohort study. Anesth Analg. 2019;128(6):1129–36. . 10.1213/ANE.0000000000004028 31094777

[11] Saager L, Maiese EM, Bash LD, Meyer TA, Minkowitz H, Groudine S, Philip BK, Tanaka P, Gan TJ, Rodriguez-Blanco Y, Soto R, Heisel O. Incidence, risk factors, and consequences of residual neuromuscular block in the United States: The prospective, observational, multicenter RECITE-US study. J Clin Anesth. 2019;55:33–41. Epub 2018 Dec 27. PMID: 30594097. 10.1016/j.jclinane.2018.12.042 30594097

[12] Crosby P, Smolkin C, Sanicola C, Rosenbluth A, Pryor A, Swaszek L, Fuller S, Spaniolas K. Effect of Sugammadex on Post-operative Urinary Retention After Bariatric Surgery. Obes Surg. 2025;35(3):823–8. 10.1007/s11695-025-07705-y. Epub 2025 Feb 12. PMID: 39934519. 39934519

[13] Bamdad MC, Brown CS, Kamdar N, Weng W, Englesbe MJ, Lussiez A, Patient. Surgeon, or Hospital: Explaining Variation in Outcomes after Colectomy. J Am Coll Surg. 2022;234(3):300–9. PMID: 35213493; PMCID: PMC10369366. 10.1097/XCS.0000000000000063 PMC10369366 35213493

